# Coping styles, strategies and psychological distress amongst perinatal individuals during the COVID-19 pandemic: a rapid review

**DOI:** 10.3389/fgwh.2025.1666741

**Published:** 2025-11-13

**Authors:** Alissa Papadopoulos, Emma G. Duerden

**Affiliations:** 1 Applied Psychology, Faculty of Education, Western University, London, ON, Canada; 2 Western Institute for Neuroscience, Western University, London, ON, Canada; 3 Neuroscience, Schulich School of Medicine and Dentistry, Western University, London, ON, Canada; 4 Psychiatry, Schulich School of Medicine and Dentistry, University of Western Ontario, London, ON, Canada

**Keywords:** perinatal, distress, coping, COVID-19, depression, anxiety

## Abstract

**Introduction:**

Perinatal individuals are at an increased risk of experiencing psychological distress, which often manifests in a combination of co-occurring symptoms of anxiety, depression, and stress. During the COVID-19 pandemic, the rates of psychological distress experienced by perinatal women dramatically increased, in some cases doubling or even tripling. This increase is concerning as psychological distress can impact the health and wellbeing of mothers and their offspring, including an offspring's neurocognitive, physical, mental, and socio-emotional development. The strategies a perinatal individual uses to cope with psychological distress are modifiable and, therefore, can be targeted to help improve outcomes for mothers and their offspring.

**Methods:**

This rapid review describes and synthesizes the literature related to coping with perinatal psychological distress during the COVID-19 pandemic. This review included twenty-four cross-sectional studies.

**Results:**

Perinatal individuals reported using various coping strategies to deal with the COVID-19 pandemic, including social strategies (e.g., connecting with others); physical strategies (e.g., exercising); cognitive strategies (e.g., positive re-appraisal); and spiritual strategies (e.g., prayer). An avoidant style of coping and its accompanying behaviours, including disengagement, substance use, and distraction via screen time/social media use, were significantly associated with higher levels of psychological distress. Strategies associated with lower levels of psychological distress included sleep and social support.

**Discussion:**

Future studies should address the impact of technology on coping and the long-term impact of coping styles used during the COVID-19 pandemic on the wellbeing of mothers and their offspring. Although this rapid review centered on the COVID-19 context, its findings are broadly relevant to women worldwide who continue to experience prolonged stressors such as climate change, poverty, and conflict.

## Introduction

Perinatal psychological distress describes the presence of emotional distress manifesting as symptoms of depression, anxiety, and stress from pregnancy through 1-year after delivery (1). The symptoms of depression, anxiety, and stress can often co-occur and can sometimes reach clinical significance, warranting a diagnosis (1, 2). Compared to the general population, perinatal individuals are at an increased risk of experiencing psychological distress, likely due to the marked physical, emotional, and social changes that are characteristic of the perinatal period (3). Evidence suggests that perinatal psychological distress is related to a lower quality of life and poorer physical health in those affected (4, 5).

The prevalence of psychological distress amongst perinatal individuals has significantly increased during the COVID-19 pandemic (6). Compared to pre-pandemic levels, symptoms of stress, depression, and anxiety were all found to dramatically increase amongst perinatal individuals around the world during the COVID-19 pandemic (6–14).

Perinatal psychological distress not only affects the wellbeing of mothers, but it can also negatively impact their offspring. The literature suggests that both prenatal and postpartum maternal psychological distress is associated with adverse outcomes in offspring. These include low birth weight (15), altered fetal, infant, and childhood brain development (16–18), adverse cognitive, socio-emotional, and motor outcomes (19), and an increased risk of neurodevelopmental and mental health disorders (17, 19).

Studies conducted during the COVID-19 pandemic, specifically, have documented altered infant outcomes related to perinatal psychological distress. These include altered infant brain structure (20–22), functional brain connectivity (20) and development, including differences in motor- (23), socioemotional- (24, 25), and cognitive- (22) functioning.

The research on the effects of perinatal psychological distress during the COVID-19 pandemic has led to identifying some modifiable risk- and protective factors (20, 23). For example, Papadopoulos and colleagues (23) found that during the COVID-19 pandemic, the duration of psychological distress in mothers was a risk factor for adverse infant motor development. Specifically, the 2-month-old infants who were most at risk of motor impairment were those whose mothers were depressed during both pre-and post-natal periods. In addition, social support was observed to protect infants from the negative impacts of maternal perinatal psychological distress during the COVID-19 pandemic (20). A study conducted by Manning and colleagues (20) demonstrated that perinatal distress altered the functional brain connectivity in 3-month-old infants whose mothers received low social support. Such studies emphasize the importance of identifying modifiable factors that can mitigate the negative impact that psychological distress has on perinatal individuals and their offspring.

One crucial, modifiable factor is coping - including different coping styles and strategies. Generally, avoidant coping styles, including strategies such as denial, distraction, substance use, and disengagement, are ineffective in dealing with perinatal psychological distress (26, 27). On the other hand, the efficacies of spiritual strategies and active coping styles, such as problem-focused coping (planning, information, and positive appraisal) and emotion-focused coping (venting, self-blame, acceptance, humour, religion, and emotional support) are variable, with their effectiveness being dependent on one's current situation (26, 27).

The COVID-19 pandemic was an unprecedented, worldwide, and chronic stressor accompanied by many restrictions that impacted lives on a day-to-day basis (27). Therefore, it is essential to examine coping among perinatal individuals within the context of the COVID-19 pandemic, as the insights gained can inform future guidelines for managing psychological distress in response to disaster-related and other long-term stressors. Although this review focuses on pandemic-related coping, the findings have broader relevance for women globally who face ongoing challenges such as climate change, poverty, and conflict. Identifying adaptive coping mechanisms is urgently needed to support the mental health and wellbeing of both mothers and their offspring across diverse and prolonged stress contexts. Although there is a substantial body of literature regarding coping and perinatal psychological distress during the COVID-19 pandemic, it has yet to undergo a structured review. Therefore, this rapid review aims to describe and synthesize the literature related to perinatal coping strategies, styles, and perinatal psychological distress during the COVID-19 pandemic by answering two questions: What coping strategies and styles did perinatal individuals use during the COVID-19 pandemic?; What is the relationship between psychological distress (depression, anxiety, stress) and the different coping styles/strategies used by perinatal individuals during the COVID-19 pandemic?

## Methods

### Search strategy

On July 8, 2024, the PsycINFO (ProQuest), PubMed, Scopus, Web of Science, and Google Scholar databases were searched for relevant literature. The search terms used included various combinations of terms related to coping (“coping,” “coping techniques,” “ coping styles,” “resilience,” and “Brief COPE”), the perinatal period (“pregnancy,” “pregnant,” “postpartum,” and “perinatal”), psychological distress (“mental health,” “depression,” “stress,” “anxiety,” “distress,” and “psychological distress”), and the COVID-19 pandemic (“COVID-19,” and “pandemic”) with Boolean operators (“AND”). The search filters included: 2020–2024, English, full-text, and peer-reviewed.

### Selection criteria

The inclusion criteria were full-text peer-reviewed studies with primary data collection from 100 or more participants published between 2020 and 2024. The target population was perinatal individuals (pregnant to 12- months postpartum) at any point during the COVID-19 pandemic (from March 11, 2020, to May 5, 2023). The target topics for the studies were those that focused on coping styles, coping strategies and the relationship between coping styles and strategies and psychological distress —stress, anxiety, and depression. The stress, anxiety, and depression did not need to be directly related to COVID-19; rather, the distress simply had to occur during the COVID-19 pandemic period.

The exclusion criteria were books, editorials, dissertations, protocol documents, case reports, and reviews (rapid, systematic, meta-analyses). Studies were also excluded if participants in the sample had a serious health condition (e.g., HIV, Hepatitis) or were over 12 months postpartum, and if distress was measured in terms other than stress, anxiety, or depression (e.g., trauma).

### Data extraction & synthesis

One author (A.P.) extracted data from all the studies included in the final review. The data extracted from each study included author names, year of publication, country of origin, participant information, study design, sampling methods, study instruments, and relevant results. One author (A.P.) employed a narrative style to synthesize the results from the 24 papers included in the final review.

### Quality & risk of bias assessments

One author (A.P.) thoroughly evaluated the quality of the evidence and the risk of bias for each of the 24 studies included in the final review. The Joanna Briggs Institute's (JBI) cross-sectional and qualitative critical appraisal tools were used to evaluate evidence quality (28). The cross-sectional critical appraisal tool, consisting of 8 questions, was used to evaluate the quantitative and mixed-methods studies. A score of 7 or higher on the cross-sectional critical appraisal tool indicates high-quality evidence (28). The qualitative critical appraisal tool, consisting of 10 questions, was used to assess the evidence quality of the single qualitative study included in the final review (28). A score of 9 or higher on the qualitative critical appraisal tool indicates high-quality evidence (28).

A modified protocol from McMaster's CLARITY group was used to evaluate the risk of bias in the studies (29). The risk of bias was assessed by examining the representativeness of the participant sample, the validity of the study instruments used, and, if available, the studies' response rates and amounts of missing data. Indicators of a high risk of bias included non-probabilistic sampling methods, a lack of evidence regarding the reliability and validity of the study instruments, a response rate under 75%, or a missing data rate of more than 15% (29).

## Results

### Search results

**Figure 1:**
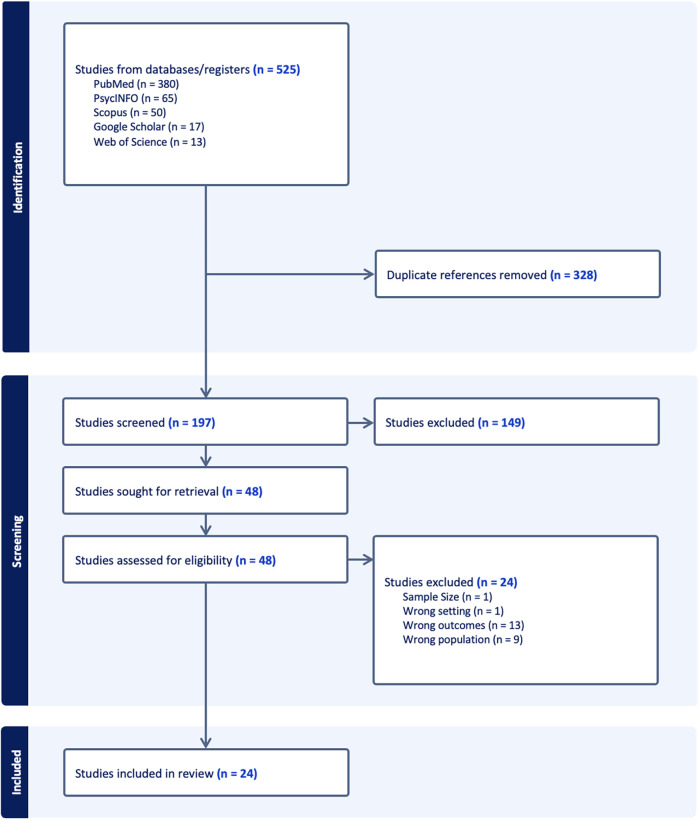
Study selection process PRISMA flowchart.

The initial search yielded 525 studies, and 328 duplicates were removed. One author (A.P.) screened the titles and abstracts of the 197 remaining studies and removed 149 for failing to meet the selection criteria (outlined in the methods section). One author (A.P.) retrieved and reviewed the full texts of the remaining 48 studies. A total of 24 studies met the eligibility criteria and were selected to be included in the rapid review. See Figure 1 includes a PRISMA flowchart outlining the search process and results.

### Data extraction

The extracted data are presented below in Table 1. The last row of Table 1 explains the abbreviations used in the study instruments column.

**Table 1:** Summary of reviewed studies. Coping strategies & styles and their relationship with psychological distress in perinatal individuals during the COVID-19 pandemic.

Authors & publication year	Country of origin	Participants	Data collection period	Study design & sampling	Study instruments	Results
Abdus-salam et al., 2022 (30)	Nigeria	380 pregnant individuals	August – November 2020	Cross-sectional; Quantitative; Simple random sampling from antenatal clinic	Multiple choice question regarding coping strategies	The most frequently reported coping strategies amongst the sample were spiritual & social support (36%). The coping strategy reported the least was using social media (2.7%).
Badon et al., 2022 (31)	United States	8,320 pregnant individuals	June 2020 – May 2021	Cross-sectional; Quantitative; Purposive convenience sampling from healthcare centre	PHQ-8; GAD-7; Multiple choice questions regarding coping strategies	The coping strategies that were associated with a lower prevalence of moderate to severe symptoms of anxiety (16%–34%) and depression (29%–38%) included engaging in physical exercise, family activities, and talking with others. Coping strategies that were associated with increased prevalence of moderate to severe symptoms of anxiety (65%–84%) and depression (66%–86%) included increased frequency of screen time, eating, and talking with healthcare providers.
Barbosa-Leiker et al., 2021 (32)	United States	162 total individuals: 125 pregnant & 37 within 1st year after delivery	April – June 2020	Cross-sectional; Mixed Methods; Cluster sampling of perinatal women in the United States	ISEL; Multiple choice question regarding coping strategies	Postpartum individuals were significantly more likely than pregnant individuals to use the following coping strategies: taking media breaks (*p* < 0.001), engaging in healthy behaviours, such as exercise, eating healthy, sleep, and avoiding substances (*p* = 0.006), taking time to relax (*p* = 0.027), & connecting with others (*p* = 0.004). Individuals with lower incomes were significantly less likely to engage in adaptive coping behaviours, such as connecting with others (*p* = 0.047) and self-care practices, including breathing, stretching & meditation (*p* = 0.007) compared to those with higher incomes. Individuals with lower incomes and individuals of colour reported significantly less overall social support than those with higher incomes (*p* = 0.012) and those who identified as White (*p* = 0.007).
Bayrampour & Tsui, 2023 (33)	Canada	268 individuals at 4-months postpartum	March 2020 – April 2021	Cross-sectional; Qualitative; Purposive convenience sampling from healthcare centres	Open-ended question regarding coping strategies	The coping strategies reported by the sample included online support groups, staying connected with others, exercise, alone time, outdoor time, meditation, and drinking alcohol.
Crowe & Sarma, 2022 (34)	Ireland	761 pregnant women	January 2021	Cross-sectional; Quantitative; Purposive convenience sampling using social media	Brief COPE; PSS	Higher levels of avoidant style coping significantly predicted higher levels of perceived stress (*p* < 0.001).
Davis et al., 2021 (35)	Australia	174 total individuals: 31 pregnant & 143 postpartum up to 1st year after delivery	June – July 2020	Cross-sectional; Mixed Methods; Purposive sampling from larger study cohort	SCS; MAAS; MHC-SF; PSS	Higher use of mindfulness and self-compassion significantly predicted better mental health and lower perceived stress levels (all *p* < 0.001).
Dib et al., 2020 (36)	United Kingdom	1,329 individuals with infants up to 1st year after delivery.	May – June 2020	Cross-sectional; Quantitative; Purposive convenience sampling using social media	Multiple choice questions for frequency of coping strategy use & mental health	Utilizing peer support groups (e.g., breastfeeding groups, mother-baby groups, etc.) predicted lower levels of mental health problems (anxiety, low mood, and loneliness), but this trend did not reach significance (*p* = 0.056).
Dol et al., 2023 (37)	Canada	331 individuals within 6- months after delivery	March – October 2020	Cross sectional; Mixed Methods; Purposive convenience sampling using social media	Open-ended question regarding coping strategies	The coping strategies reported by the sample included receiving support from others, bringing others into their COVID-19 “bubble,” connecting using technology (video calls, online support groups), exercise, orientation towards the present moment, and self-reassurance that the pandemic would pass.
Firouzbakht et al., 2022 (38)	Iran	318 pregnant individuals	April – May 2021	Cross-sectional; Quantitative; Purposive convenience sampling from healthcare centres	EPDS; CDA-Q; Brief COPE	An avoidant coping was the most utilized amongst the sample, and its use significantly predicted higher levels of depressive symptoms (*p* = 0.046).
Goldstein et al., 2023 (39)	United States	616 total individuals: 303 were pregnant & 313 within 6-months after delivery	August – November 2020 & January – April 2021	Cross-sectional; Quantitative; Purposive convenience sampling from healthcare centres	EPDS; Multiple choice questions for perceived stress severity & coping strategies	Perinatal individuals who screened positive for clinically-elevated depression, anxiety and stress at the highest rates reported high levels of risk factors (i.e., lack of companionship, isolation, need for/access to mental health services) and low levels of protective factors (i.e., adequate sleep, healthy eating, social interaction, social support).
Gómez-Baya et al., 2022 (40)	Spain	3,356 total individuals: 1,402 pregnant & 1,954 postpartum up to 6-months after delivery	June 2020 – June 2021	Cross-sectional; Quantitative; Purposive convenience sampling using social media	GAD-7; EDPS; COPE-IS	In pregnant and postpartum individuals, sleeping well at night significantly predicted lower levels of anxiety and depression. Talking to a mental health professional significantly predicted higher levels of anxiety and depression in pregnant individuals and higher levels of anxiety in postpartum individuals (all *p* < 0.001).
Han et al., 2022 (41)	China	969 pregnant individuals	December 2021 – April 2022	Cross-sectional; Quantitative; Purposive convenience sampling from hospitals	CAQ; SCSQ	Nulliparous pregnant individuals used significantly more “positive coping strategies,” such as problem-solving & positive appraisal (*p* = 0.025) and more “negative coping strategies,” such as substance use & avoidance (*p* = 0.005) than multiparous pregnant individuals. A positive coping style significantly predicted less fear regarding childbirth, and a negative coping style significantly predicted more fear (both *p* < 0.000).
Khoury et al., 2021 (42)	Canada	304 pregnant individuals	June – July 2020	Cross-sectional; Quantitative; Purposive, convenience sampling through social media	CES-D; GAD-7; CWS; PSS; Brief COPE	Dysfunctional coping (denial, disengagement, self-blame) was significantly predictive of worse mental health (anxiety, depression, worry, perceived stress, sleeping problems) (*p* < 0.01). Emotion-focused coping was significantly predictive of better mental health (*p* < 0.01).
Kiliç et al., 2022 (43)	Turkey	191 pregnant individuals	November 2020 – February 2021	Cross-sectional; Quantitative; Purposive convenience sampling from obstetric outpatient clinic	STAI; Brief COPE; MSPSS	Individuals with clinically elevated levels of state- and trait-anxiety used behavioural disengagement more than individuals below clinical cut-offs (*p* < 0.05, *p* < 0.0001). Individuals with clinically elevated trait-anxiety vented emotions and used substances significantly more than those below cut-offs (*p* < 0.001, *p* < 0.05) and had significantly lower levels of perceived family support (*p* < 0.05). Individuals below state-anxiety clinical cut-offs used acceptance (*p* < 0.05) and positive appraisal (*p* < 0.01) significantly more than those who were clinically elevated and had significantly higher scores on all measures of social support (family, friends, significant other, total) (*p* < 0.001 - *p* < 0.05). More use of disengagement (*p* = 0.009) and venting emotions (*p* = 0.001) significantly predicted higher levels of trait-anxiety.
Kinser et al., 2021 (44)	United States	524 total individuals: 306 pregnant & 218 postpartum up to 6-months after delivery	April – June 2020	Cross-sectional; Mixed-Methods; Purposive convenience sampling from hospital system	BSI-18; COPE-IS; open-ended questions regarding coping strategies	Coping strategies reported by the sample included talking with other mothers, using technology to keep in touch with others, limiting social media/news content, gratitude, acceptance, accessing mental health resources, self-care, going outdoors, exercising, eating healthy, mindfulness/meditation, prayer, and accepting help from others. Getting a good night's sleep significantly predicted lower levels of both depression and anxiety (*p* = 0.0003, *p* = 0.0201). Spending less time watching the news significantly predicted lower anxiety levels (*p* = 0.0197). Higher use of comfort foods as a coping strategy significantly predicted higher levels of both depression and anxiety (*p* = 0.0106. *p* = 0.0139). Using more social media significantly predicted higher levels of depression (*p* = 0.0139), and helping others significantly predicted higher levels of anxiety (*p* = 0.0096).
Levinson et al., 2023 (27)	United States	7,383 pregnant individuals	April – May 2020 & December 2020	Cross-sectional; Quantitative; Purposive convenience sampling from larger study cohort	NuPCI; GAD-7; PHQ-2; PREPS; NuPDQ	The sample used positive appraisal the most (*p* < 0.001) and avoidant style coping strategies the least (*p* < 0.001). The use of spiritual and avoidant coping methods significantly predicted higher levels of mood/anxiety symptoms, whereas positive appraisal methods predicted lower mood/anxiety symptoms (all *p* < 0.001).
LoGiudice & Bartos, 2022 (45)	United States	185 pregnant individuals	March – May 2020	Cross-sectional; Mixed Methods; Snowball sampling through social media	Open-ended prompt to describe experience during pandemic	Gratitude and self-reassurance that the pandemic will eventually resolve were coping strategies reported by individuals in the sample.
Mollons et al., 2024 (46)	Canada	336 pregnant individuals that self-identified as Indigenous	April 2020 – April 2021	Cross-sectional; Mixed Methods; Purposive convenience sampling from larger study cohort	SSEQ; ISEL; EPDS; PROMIS; Open-ended questions regarding coping strategies	Self-reported coping strategies included being outdoors, exercising, walking, self-care, being creative, cultural practice, connecting with others in-person and virtually, connecting with pets, limiting news exposure, creating structured routines, accessing therapy, mindfulness, and gratitude. Having more social support from one's partner significantly predicted lower levels of depression (*p* < 0.001).
Penengo et al., 2022 (47)	Italy	325 pregnant individuals	December 2020 – June 2021	Cross-sectional; Quantitative; Purposive convenience sampling from hospital	NuPDQ; NuPCI; PREPS; GAD-7; PHQ-2	Individuals with clinically-elevated anxiety used avoidance style and spiritual coping strategies significantly more than those below the clinical cut-off (*p* < 0.001). Individuals with clinically-elevated depression used avoidance significantly more than those below the clinical cut-off (*p* < 0.001).
Preis et al., 2020 (48)	United States	4,451 pregnant individuals	April – May 2020	Cross-sectional; Quantitative; Purposive convenience sampling through social media	PREPS	Having access to outdoor space significantly predicted lower levels of perinatal infection stress (concerns about contracting COVID-19) and preparedness stress (feeling unprepared for birth during the pandemic) (both *p* < 0.001). Practising healthy behaviours (e.g., good sleep, diet, exercise) significantly predicted lower levels of Preparedness stress (*p* < 0.001).
Rimal et al., 2022 (49)	Nepal	115 pregnant individuals	May – July 2020	Cross-sectional; Quantitative; Purposive, convenience sampling from obstetric care centre	Brief COPE; CPDI	Problem-focused (*p* < 0.001), emotion-focused (*p* = 0.015), and dysfunctional (distraction, venting behavioural disengagement, denial, self-blame, substance use) (*p* = 0.035) styles of coping showed significant positive correlations with psychological distress. Problem-focused coping had the strongest correlation with psychological distress (r = 0.371).
Spinola et al., 2020 (50)	Italy	243 postpartum individuals within 1st year after delivery	May – June 2020	Cross-sectional; Quantitative; Purposive, convenience sampling through social media & postpartum groups	EPDS; PSS; Brief COPE; MSSS	Avoidant-style coping showed a significant, positive, low-strength correlation with depression (r = 0.356, *p* = 0.001). The study found regional differences in coping strategy use: Pregnant individuals in Northern Italy used substances and self-distraction as coping strategies at significantly higher rates than those from South to Central Italy (*p* = 0.01, *p* = 0.04).
Timircan et al., 2021 (51)	Romania	304 total pregnant individuals: 168 COVID-positive & 136 COVID-negative	April – May 2021	Cross-sectional; Quantitative; Purposive, convenience sampling from healthcare centres	COPE-60	COVID-positive individuals used disengagement significantly more than those who were COVID-negative (*p* = 0.048). COVID-negative individuals used an engagement coping style (positive reinterpretation, religion, assistance for tangible needs, venting, emotion-focus, social and emotional support, acceptance, planning) significantly more than those who were COVID-positive (*p* = 0.007).
Werchan et al., 2022 (52)	United States	4,412 total individuals: 2,876 were pregnant & 1,536 within 1st year after delivery	March – October 2020	Cross-sectional; Quantitative; Purposive, convenience sampling from research institutions	BSI-18; Multiple choice questions regarding coping strategies	Pregnant & postpartum women adopting a “vegging out” coping style (use of screen time, social media, and comfort foods) reported significantly higher levels of anxiety and global psychological distress compared to those using other coping styles (both *p* < 0.05). Pregnant women using “vegging out” techniques to cope also reported significantly higher levels of depression (*p* < 0.05). Postpartum women using “vegging out” techniques to cope accompanied by low levels of self-care (sleep, meditation, healthy eating, etc.) and social support reported significantly higher levels of depression (*p* < 0.05).

### Study characteristics results

For detailed information regarding study characteristics, including countries of origin, study design, sampling methods, participants, data collection periods, and study instruments, refer to Table 1.

#### Countries

The studies used in this rapid review were from 13 countries of origin. Eight studies were from the United States, four were from Canada, two were from Italy, and single studies were from Nigeria, Ireland, Australia, the United Kingdom, Iran, Spain, China, Turkey, Nepal, and Romania.

#### Study design

All the studies included in the final review employed a cross-sectional study design. Six studies used mixed methods (quantitative & qualitative), 17 studies used quantitative methods, and one study used qualitative methods.

#### Sampling methods

Most studies used purposive, convenience sampling techniques to identify eligible individuals online or within healthcare centres. One study used simple random sampling to select individuals from an antenatal clinic to take part in the study (30), one study used snowball sampling methods to recruit individuals through social media (45), and one study used cluster sampling (32).

#### Participants

The total number of participants across all 24 studies was 35,757. In the mixed methods studies, the participant samples ranged between 162 (32) and 336 participants (46). In the quantitative studies, the participant samples ranged between 115 (49) and 8,320 participants (31). The sample size of the single qualitative study was 268 participants (33).

#### Data collection period

All studies collected data between 2020 and 2022. Thirteen studies collected data exclusively in 2020, ranging from March through December. Seven studies collected data from 2020 through 2021, ranging from April 2020 through June 2021. Three studies collected data exclusively in 2021, ranging from January through May. One study collected data from December 2021 through April 2022.

#### Study instruments

All the studies used self-report measures to collect data regarding the participants coping strategies, coping styles, and levels of psychological distress, including anxiety, stress, and depression.

##### Measures of coping

Six studies used multiple-choice questions to gather information on the coping strategies used by participants. Five studies used open-ended questions to ask participants to describe how they coped during the COVID-19 pandemic. The standardized measure of coping that was used most often was the Coping Styles Scale Brief Form (Brief COPE), and it was used in six studies. The Brief COPE is a 28-question, standardized, self-report measure of coping styles and strategies (53). The questions include information about receiving emotional support, watching TV, religious practices, acceptance, and beyond (53). Participants rate the frequency they use each coping strategy on a 4-point Likert scale from 1, “I haven't been doing this at all,” to 4, “I’ve been doing this a lot” (53). The items on the Brief COPE load onto three different subscales that represent different coping styles, including avoidant, problem-focused, and emotion-focused coping styles (53). The avoidant coping style is characterized by self-distraction, denial, substance use, and disengagement (53). The problem-focused coping style is characterized by planning and positive reframing, and the emotion-focused coping style is characterized by self-blame, acceptance, humour, venting, receiving emotional support, and religious practices (53). Other standardized measures of coping that were used by either 1 or 2 studies included in the review were the COPE-IS, NuPCI, ISEL, SCS, MAAS, SCSQ, MSPSS, SSEQ, MSSS, and COPE-60.

##### Measures of psychological distress

Six of the studies included in the final review focused only on coping styles and strategies and did not include psychological distress as an outcome measure (30, 32, 33, 37, 45, 51). Of the remaining eighteen studies, three used stress to measure psychological distress (34, 35, 48), two used anxiety (41, 43), and the remainder used some combination of depression, anxiety, and stress.

The standardized measure of anxiety that was used most often was the General Anxiety Disorder-7 (GAD-7), and it was used in five studies. The GAD-7 is a self-report measure that assesses symptoms of general anxiety disorder that an individual has experienced in the last two weeks (54). The GAD-7 includes seven items that are rated on a 4-point Likert scale, assessing the frequency that each symptom is experienced from 0, “not at all,” to 3, “nearly every day” (54). Scores above 4 indicate mild anxiety, scores above 9 indicate moderate anxiety and scores above 15 indicate severe anxiety (54). The GAD-7 is a useful scale for perinatal populations (55).

The standardized measure of depression that was used most often was the Edinburgh Postnatal Depression Scale (EPDS), and it was used in four studies. The EPDS is a self-report measure used in perinatal populations to measure symptoms of depression experienced in the week prior (56). The EPDS includes ten items that are rated on a 4-point Likert scale that assesses the frequency that each depressive symptom is experienced from 0, “not at all/never”, to 3, “quite often, most of the time, etc.” (56). Scores of 13 or more indicate that depression might be present (56).

The standardized measure of stress that was used most often was the PSS, and it was used in four studies. The PSS is a self-report measure that assesses the frequency of perceived stress symptoms in the past month using ten items rated on a 5-point Likert Scale ranging from 0, “never,” to 4, “very often” (57). Perceived stress indicates how much an individual feels stress subjectively (57). Scores 14 and above indicate moderate perceived stress, and scores 27 and above indicate high perceived stress (57). The PSS has been validated in perinatal samples (58).

Other psychological stress measures used in 1–3 studies include the BSI-18, PHQ, PREPS, STAI, CAQ, CDA-Q, CES-D, CPDI, CWS, MHC-SF, and PROMIS. Additionally, some studies used un-standardized measures to evaluate psychological distress, such as multiple-choice questions.

### Quality & risk of bias assessment results

Of the 17 quantitative and six mixed-methods studies included in the review, 12 were determined to provide high-quality evidence (scoring seven or higher on the JBI cross-sectional study appraisal tool). Of the 11 quantitative and mixed methods studies that did not meet the high-quality evidence cut-off, most scored between 5 and 6, which indicates fair quality evidence, with two studies scoring 3–4, which indicates lower quality evidence. The studies below the high-quality evidence cut-off mostly lost points for lacking a detailed account of participant inclusion criteria and for lacking evidence regarding the reliability and validity of their study instruments. For example, measuring coping and psychological distress using measures, such as open-ended or multiple-choice questions that were not standardized or validated prior to use. The single qualitative study was deemed to be high quality (scoring above 9) according the JBI qualitative study appraisal tool. A detailed breakdown of the critical appraisal of evidence quality is in Appendix A.

All the studies included in the review were determined to have a high probability of being biased due to a lack of representativeness in the participant samples. The samples tended to mainly capture individuals with high levels of education and high socioeconomic status. The study samples also tended to lack ethnic and racial diversity, with many study samples being predominantly White. In addition, the sampling methods used by all, but one study were non-probabilistic. Many studies used online recruitment methods, had participants complete surveys online, and some studies used open-ended questions or multiple-choice measures without testing their psychometric properties, which all increased the risk of bias. A detailed breakdown of the results of the risk of bias assessment can be found in Appendix B.

### Coping strategies & styles results

This rapid review aimed to answer the question: “What coping strategies and styles did perinatal individuals use during the COVID-19 pandemic?” The following results were observed.

Overall, perinatal individuals used a wide range of coping strategies and styles throughout the COVID-19 pandemic. One author (A.P.) grouped the main coping strategies and styles reported into four categories: social, cognitive, physical, and spiritual/cultural.

An important social strategy reported by perinatal individuals was receiving support from others, including social- (30, 39, 43, 46, 51, 52), instrumental- (44, 49, 51), and emotional- support (42, 51). Staying connected with others was another critical coping strategy reported by perinatal individuals (31–33, 36, 37, 39, 44). Further, perinatal individuals also reported connecting with other pregnant and postpartum mothers via online support groups (33, 36, 44), and some connected with mental health/healthcare professionals (31, 39, 40, 44).

The cognitive strategies perinatal individuals reported using during the COVID-19 pandemic included meditation/mindfulness (33, 35, 37, 44, 46), self-compassion (35), self-reassurance that the pandemic will pass (37, 45), positive re-framing/reinterpretation/appraisal (27, 42, 43, 49), acceptance of the current situation (42–44), and gratitude (44–46). Perinatal individuals also used disengagement for avoidance of stressors (27, 32, 34, 38, 42, 47, 49–51). Some notable examples included self-distraction from thoughts and problems via increased time spent on social media and watching television (30, 31, 44, 52) or by avoiding information overload by limiting time spent on social media or watching the news (32, 44, 46, 52).

The most common physical coping strategy reported included exercise, like walking and stretching (31–33, 37, 44, 46, 48). Other strategies included eating, whether it be healthy eating (32, 39, 44) or eating comfort foods (31, 52) and sleep (32, 39, 40, 42, 44, 48, 52). Some individuals reported avoiding substances such as drugs and alcohol (32), and some reported using these substances as a coping strategy (34, 43, 49–51).

Lastly, some individuals used spiritual or cultural practices as a coping mechanism during the COVID-19 pandemic (27, 44, 46, 47). In one study from Nigeria, spiritual coping was reported as the most frequently used strategy among the pregnant individuals in the sample (30). Spending time alone (33) and spending time outdoors (31, 44, 46, 48) were also reported and classified here as spiritual coping strategies.

#### Comparisons: perinatality, income, race, parity, region & COVID

One study compared coping between pregnant and postpartum individuals and between individuals of races and income levels (32). This study found that postpartum individuals were significantly more likely than pregnant individuals to take breaks from social media *p* < 0.001), engage in healthy behaviours (exercise, eating healthy, sleep, avoiding substances) (*p* = 0.006), take time to relax (*p* = 0.027), & connect with others (*p* = 0.004). They also found that individuals with higher income levels were significantly more likely to engage in adaptive coping behaviours such as connecting with others (*p* = 0.047) and self-care practices such as breathing, stretching, and meditation (*p* = 0.007). Individuals with higher income levels also had more access to social support than those with lower incomes (*p* = 0.012). Finally, they found that non-Hispanic White individuals had significantly higher levels of social support than individuals of other races (*p* = 0.0007).

One study found that pregnant individuals with no other living children made significantly more use of coping strategies in general than individuals with one or more living children (41). They found this was true for coping strategies they termed to be both “positive” (problem-solving and positive appraisal) (*p* = 0.025) and “negative” (substance use, avoidance) (*p* = 0.005) (41).

A study conducted in Italy with 325 pregnant individuals found a significant difference in coping strategies between individuals residing in different regions of Italy (50). Pregnant individuals in Northern Italy used substances and self-distraction significantly more than those in Central/Southern Italy (*p* = 0.01, *p* = 0.04) (50).

Finally, one study compared coping between COVID-positive and COVID-negative pregnant individuals (51). They observed that COVID-positive individuals used an avoidant style of coping (denial, substance use, mental and behavioural disengagement) significantly more than COVID-negative individuals (*p* = 0.048) and used an engagement coping style (positive reinterpretation, humour, religion, assistance for tangible needs, venting, emotion-focus, social and emotional support, acceptance, planning) significantly less (*p* = 0.007) (51).

### Psychological distress, coping styles & strategies results

This rapid review also aimed to answer the question: “What is the relationship between psychological distress (depression, anxiety, stress) and the different coping styles/strategies used by perinatal individuals during the COVID-19 pandemic?” The following results were observed.

#### Coping styles & psychological distress

The coping styles that were found to be significantly associated with higher levels of psychological distress included an avoidant-coping style (27, 34, 38, 42, 43, 47, 49, 50) and a problem-focused coping style (49). There were conflicting results regarding the association between psychological distress and an emotion-focused coping style. A study of 304 pregnant individuals conducted in Canada found an emotion-focused coping style to be associated with lower levels of distress via mediation analysis (42); however, using correlation analysis, a study conducted in Nepal of 115 pregnant individuals found emotion-focused coping to be associated with higher levels of distress (49).

#### Coping strategies & psychological distress

On a finer scale, the individual coping strategies that were found to be significantly associated with lower levels of psychological distress included physical exercise (31, 48), connecting with others (31), getting a good night's sleep (40, 44, 48), eating healthy (48), positive appraisal (27, 43), social support (43, 46), spending less time watching the news (44), mindfulness (35), self-compassion (35), and going outdoors (48).

The coping strategies that were found to be significantly associated with higher levels of psychological distress included increased screen time/social media use (31, 44, 52), eating comfort foods (31, 44, 52), talking with mental health/healthcare providers (31, 40), religious/spiritual practices (27, 47), venting emotions (43), the use of substances (43), disengagement (43), and helping others (43).

## Discussion

This rapid review aimed to describe and synthesize the literature on coping strategies and styles and their relationship with psychological distress in perinatal individuals during the COVID-19 pandemic. A rapid review methodology was selected to promptly consolidate and synthesize findings. Although the acute phase of the pandemic has concluded, women globally are continually exposed to long-term, high-stress situations such as climate change, economic instability, displacement, and conflict. The COVID-19 pandemic, also a prolonged and widespread stressor, offers a relevant context for understanding coping among perinatal individuals. While this review focused on pandemic-related experiences, the findings may be generalizable to other ongoing crises affecting women's mental health and wellbeing worldwide.

The findings of this review confirmed that perinatal individuals used several different coping strategies during the COVID-19 pandemic. The strategies used included those from social, cognitive, physical, and spiritual/cultural domains. They also adopted various styles, including avoidance, emotion-focused, and problem-focused coping styles. The most cited coping strategy was staying connected with others, appearing in eight studies (31–33, 36, 37, 39, 40, 44). Some individuals explained that bringing additional people into their “COVID-19 bubble” was imperative to coping (37). The next most cited strategies were physical exercise, appearing in seven studies (31–33, 37, 44, 46, 48), and social support, appearing in six studies (30, 39, 43, 46, 51, 52). Notably, in all the studies included in this rapid review, the same general coping strategies were reported and used by perinatal individuals worldwide.

High-quality evidence was insufficient to draw definitive conclusions about the relationship between psychological distress and a problem-focused coping style. However, evidence presented in this review suggests that problem-focused coping was not linked to lower levels of psychological distress for perinatal individuals during the COVID-19 pandemic. It follows that problem-focused coping, which focuses mainly on solving and planning, might not be a beneficial coping style during a situation like the COVID-19 pandemic, as individuals very much lacked control over their situations (e.g., lockdown rules, hospital regulations, etc.) and it was difficult to plan or problem-solve with frequently changing guidelines (59). However, positive appraisal as an individual coping strategy that falls under the problem-focused coping style was associated with lower levels of distress.

The evidence regarding emotion-focused coping was conflicting, although certain aspects of emotion-focused coping were found to likely be more beneficial than others, such as acceptance, and emotional support whereas other strategies such as venting were likely to be less helpful (27, 42, 43, 51).

The review yielded a large body of evidence with eight studies suggesting an avoidant-style coping was associated with higher levels of psychological distress in perinatal individuals around the world during the COVID-19 pandemic. Of the eight studies, seven were rated as high quality in the critical appraisal (see Appendix A). The body of evidence for this coping style in relation to psychological distress included a large-scale study of 7,383 pregnant participants conducted in the United States (27), a study of 761 pregnant participants from Ireland (34), and six studies ranging between 115 and 325 pregnant participants from Iran, Canada, Turkey, Italy, and Nepal (38, 42, 43, 47, 49). One additional study included 243 postpartum participants from Italy (50). The finding that avoidant-style coping was associated with higher levels of psychological distress is in line with literature regarding coping-styles and perinatal individuals before the COVID-19 pandemic (26, 27).

It also follows that coping behaviours that are generally associated with an avoidant-style of coping, such as eating comfort foods, disengagement, using substances, and screen time/using social media, were associated with higher levels of psychological distress (31, 43, 44, 52). Many perinatal individuals used avoidant-style coping strategies to try and cope with psychological distress during the COVID-19 pandemic. The frequent use of these strategies suggests that perinatal individuals require more support, including informational resources, to help them learn about and employ alternative, more beneficial strategies for coping. An interesting finding from comparison studies was that individuals in difficult but temporary situations used avoidant coping strategies more. For example, avoidant strategies were used more often by individuals who had an ongoing infection with the COVID-19 virus (51) and by individuals living in Northern Italy, where for some time, the impact of COVID-19 was large, with hospital systems being overwhelmed, large death tolls, and a high risk of contagion (50). While avoidant-style coping strategies might not be recommended for long-term distress, the literature suggests that some avoidant-style strategies may be warranted for use on a short-term basis and could be beneficial as a harm-reduction tool (i.e., using distraction to stop the use of more harmful behaviour, like using substances) (26, 50, 51).

The individual coping strategies that are likely to be the most beneficial, as they had a good amount of high-quality evidence (see Appendix A) regarding their relationship with lower levels of psychological distress included getting a good night's sleep (40, 44, 48), positive appraisal (27, 43), and social support (43, 46). An unexpected finding was that talking with mental health/healthcare providers was associated with higher levels of psychological distress (31, 40). However, this method of coping was primarily used by individuals with very high levels of psychological distress (31, 40). A cohort study design would have been more beneficial to better assess the use of this strategy, for example, to determine whether baseline psychological distress levels decrease when talking to a mental health professional over time. Religious practice was also associated with higher levels of psychological distress, which could also indicate that individuals with high levels of distress utilized these techniques often (27, 47). In addition, the studies included in this review did not necessarily capture the nuanced differences between positive (e.g., trust in God, comfort through reading scripture, etc.) and negative (e.g., questioning God, struggles in finding meaning, etc.) religious coping methods that could affect the way this style of coping is related to psychological distress (60).

With COVID-19 limiting the ability to engage with others in-person, the results from this rapid review suggest that many individuals used technology to cope with psychological distress. This ranged from joining online peer support groups, to engaging in video calls, to accessing social media. The evidence suggests that technology's value regarding coping is variable (30–33, 37, 44, 52). Although no direct evidence was accrued from the studies in this review, based on the evidence that social support was effective in reducing symptoms of psychological distress (43, 46), technology is likely to be helpful if used to maintain contact with and receive social support from others – via video call and online support groups. Where the evidence suggests technology is most likely not helpful, is when it is used for distraction (31, 44, 52) or when it results in an overload of negative information, such as consuming excessive COVID-19 related news programming (44).

### Limitations

All studies included in this review employed a cross-sectional design, therefore no inferences regarding causality or directionality of the relationships between coping style/strategy and psychological distress can be made. In addition, an overwhelming majority of the studies used non-probabilistic sampling methods, leading to unrepresentative samples of the population at large. The generalizability of the findings is limited as most studies had high representation of perinatal individuals with high socioeconomic statuses, high levels of education, and to those who identify as white. The different instruments used for measurement of coping and psychological distress in the studies also brings variation to the constructs and limits the result comparisons that can be made across studies. Caution was taken regarding the recommendations put forth based on the results of this rapid review. Due to the nature and limited timeframe of this rapid review, we focused on summarizing key findings across studies rather than conducting in-depth comparative analyses of differences between countries, healthcare systems, or pandemic phases, which typically require more extensive data synthesis methods such as meta-analysis.

## Recommendations & conclusions

Overall, perinatal individuals used many different strategies to cope with psychological distress during the COVID-19 pandemic. Many individuals used avoidant-style coping, and it was reliably and consistently associated with higher levels of psychological distress. This result indicates the need to focus on providing clinical support and resources for coping to those experiencing perinatal psychological distress during disaster situations. Based on the evidence provided in this review, mental health intervention efforts for perinatal individuals during disaster situations should prioritize building quality sleep habits, building positive appraisal skills (e.g., cognitive behavioural therapy), and teaching individuals how to build and maintain supportive social networks. A priority would also be to guide individuals in the use of technology, with a focus on differentiating between technology use that has evidence of being beneficial in support coping (e.g., online support groups, video calls) and use that could be detrimental (e.g., excessive exposure to news outlets). For a detailed review of the coping strategies presented in this rapid review and recommendations regarding their use during disasters and similar situations, please refer to Appendix C.

### Future directions

As the COVID-19 pandemic increased the use of and reliance on technology for many worldwide, further studies examining the role and nuance of technology in coping should be a priority. In addition, future longitudinal studies should investigate the long-term impact of different coping styles and strategies perinatal individuals used during the COVID-19 pandemic. Importantly, the insights gained from this research can extend beyond the pandemic, informing support strategies for perinatal individuals facing other prolonged or large-scale stressors, such as climate change, economic hardship, or conflict. Clinicians are encouraged to prioritize and provide increased support, time, and resources to perinatal individuals to help mitigate psychological distress not only during pandemics, but in a wide range of disaster-related and chronic stress contexts. Overall, this topic warrants a larger, comprehensive systematic review.

## Data Availability

The original contributions presented in the study are included in the article/Supplementary Material, further inquiries can be directed to the corresponding author.

## Appendix A: Critical appraisal: evidence quality

Critical appraisal of evidence quality was completed using the JBI cross-sectional and qualitative study appraisal tools.

For quantitative and mixed methods studies the scores representative of a study providing high-quality evidence is 7+ and or qualitative studies the scores representative of high-quality evidence is 9+.

**Table:**

Quantitative & mixed methods studies	Were the criteria for inclusion in the sample clearly defined?	Were the study subjects and the setting described in detail?	Was the exposure measured in a valid and reliable way?	Were objective, standard criteria used for measurem-ent of the condition?	Were confoundi-ng factors identified?	Were strategies to deal with confound-ing factors stated?	Were the outcomes measured in a valid and reliable way?	Was appropr-iate statistical analysis used?			Total score & quality rating
(30)	–	+	–	–	+	+	–	+			4, low
(31)	–	+	–	+	+	–	+	+			4, low
(32)	+	+	+	–	+	+	–	+			6, fair
(34)	+	+	+	+	+	–	+	+			7, high
(35)	+	+	+	+	–	–	+	+			6, fair
(36)	+	+	–	–	+	+	–	+			5, fair
(37)	+	+	+	+	+	+	+	+			8, high
(38)	+	+	+	+	+	+	+	+			8, high
(39)	+	+	–	–	+	+	+	+			6, fair
(40)	+	+	+	+	+	+	+	+			8, high
(41)	+	+	+	+	+	+	+	+			8, high
(42)	+	+	+	+	+	+	+	+			8, high
(43)	+	+	+	+	+	+	+	+			8, high
(44)	–	+	+	+	+	+	+	+			8, high
(27)	+	+	+	+	+	–	+	+			7, high
(45)	+	+	+	+	–	–	+	+			6, fair
(46)	+	+	–	+	–	–	+	+			5, fair
(47)	+	+	+	+	+	+	+	+			8, high
(48)	–	+	–	–	+	+	+	+			5, fair
(49)	–	–	+	+	–	–	+	–			3, low
(50)	+	+	+	+	+	+	+	+			8, high
(51)	–	+	+	+	+	+	+	+			7, high
(52)	+	+	–	–	+	–	+	+			5, fair
Qualitative study	Is there congruity between the stated philosophical perspective and the research methodology?	Is there congruity between the research methodology and the research question or objectives?	Is there congruity between the research methodology and the methods used to collect data?	Is there congruity between the research methodology and the representation and analysis of data?	Is there congruity between the research methodology and the interpretation of results?	Is there a statement locating the researcher culturally or theoretica-lly?	Is the influence of the researcher on the research, and vice- versa, addressed?	Are participants, and their voices, adequately representted?	Is the research ethical according to current criteria or, for recent studies, and is there evidence of ethical approval by an appropriate body?	Do the conclusions drawn in the research report flow from the analysis, or interpretation, of the data?	Total Score & Quality Rating
(33)	+	+	+	+	+	+	+	+	+	+	10, high

+ = 1 point.

– = 0 points.

Moola et al. (28).

## Appendix B: Critical appraisal: risk of bias

Critical appraisal of the studies for risk of bias was completed using a modified version of the McMaster CLARITY Group's Risk of Bias assessment tool.

Indicators of a high risk of bias included: non-probabilistic sampling methods, study instruments lacking established evidence of validity and reliability, response rates of less than 75%, and missing data rates of greater than 15%.

**Table:**

Studies	Was the sample representative?	Was the response rate more than 75%?	Was the missing data rate less than 15%?	Was there evidence of the validity and reliability of the study instruments?	Risk of Bias
(30)	No	N/A	N/A	No	High
(31)	No	No	No	No	High
(32)	Yes	N/A	N/A	No	High
(33)	No	N/A	Yes	Yes	High
(34)	No	N/A	N/A	Yes	High
(35)	No	No	Yes	Yes	High
(36)	No	N/A	N/A	No	High
(37)	No	N/A	Yes	No	High
(38)	No	Yes	N/A	No	High
(39)	No	No	N/A	No	High
(40)	No	N/A	N/A	Yes	High
(41)	No	N/A	Yes	Yes	High
(42)	No	N/A	Yes	Yes	High
(43)	No	N/A	N/A	Yes	High
(44)	No	No	N/A	Yes	High
(27)	No	N/a	N/A	Yes	High
(45)	No	N/A	N/A	No	High
(46)	No	N/A	No	Yes	High
(47)	No	N/A	Yes	Yes	High
(48)	No	N/A	N/A	Yes	High
(49)	No	N/A	N/A	Yes	High
(50)	No	N/A	N/A	Yes	High
(51)	No	Yes	N/A	Yes	High
(52)	No	No	N/A	No	High

N/A, not available.

EvidencePartners (29).

## Appendix C: Recommendations: coping styles & strategies

Recommendations for coping strategy use amongst perinatal individuals during natural disasters or similar situations, based on research from the COVID-19 pandemic.

**Table:**

Coping strategies	Considerations	Confidence
Getting adequate sleep, receiving social support, positive appraisal/re-interpretation, healthy eating, physical exercise (e.g., walking), and connecting with family, friends, and peers (in-person or virtually – Eg. video calls, online support groups).	Medical doctors should be consulted before the use of any type of physical exercise and for modifications to diet. In-person connection with others should be based on health and safety considerations.	*High-level confidence that the strategies are beneficial and should be recommended for use.*
Self-reassurance, mindfulness/meditation, self-compassion, acceptance, gratitude, spending time outdoors, receiving emotional-support, receiving instrumental support, taking media breaks (e.g., spending less time watching the news), religious/cultural practices, and talking with mental/healthcare providers.	Mindfulness/meditation practices might need to be modified for individuals with a history of trauma. Media can be accessed to stay informed, but not accessed in excess.	*Strategies* that *can be used, minimal risk of danger/harm.*
Planning, problem-solving, venting emotions, and a focus on helping others.	Problem-solving and planning can be helpful for problems within an individual's control.	*Strategies that are likely not helpful.*
Substances (alcohol & drugs), comfort foods, denial, disengagement, self-blame, distraction, increased time spent on social media/television.	Occasional use of short-term disengagement & distraction could potentially be helpful during situations that are temporary and out of the individual's control (e.g., illness).	*High-level confidence that the use of these strategies should be avoided.*
